# Book Reviews

**Published:** 1876-11

**Authors:** 


### Book Reviews.

[NOTE.—All works reviewed in the pages of the CHICAGO MEDICAL JOURNAL AND EXAMINER may be found in the extensive stock of W. B. KEEN, COOKE & Co., whose Catalogue of Medical Books will be sent to any address upon request.]

THE MEDICAL JURISPRUDENCE OF INSANITY, by *J. H. Balfour Browne, Esq., etc.* Second edition, with references to the Scotch and American decisions. 8vo., pp. 713. Philadelphia: Lindsay & Blakiston. 1876.

The first edition of this very valuable contribution to the literature of medical jurisprudence met with favorable consideration from its many critics. The present volume claims very justly for itself additional appreciation, not entirely based on previous merit, but because it has been in great part re-written and re-arranged, and, in some instances, its propositions have been reconsidered and recast; and, moreover, the new and important cases that have been decided, and the several works on law and insanity that have been issued since the previous publication, have been carefully considered, and so far as deemed desirable have been embodied in this edition. In addition to this its usefulness as a book of reference has been very much enhanced by the subdivision of the chapters into sections, to which the index and table of contents refer.

Without entering into a detailed description of the work, we earnestly commend it to the profession as one of the most desirable in the specialty, not only on account of the exhaustive and scientific character of his descriptions, but because of the striking originality displayed on almost every page. We endorse the book generally, but take exception to certain of its propositions, especially to the tests of responsibility. These tests are those agreed upon by the English judges after the celebrated trial of McNaughton, in 1843, in reply to questions propounded by the House of Lords. We object emphatically to the answers to the third and fourth questions. These are, *First*, "That to establish a defense on the ground of insanity, it must be clearly proved that at the time of committing the act the accused was laboring under such a defect of reason from disease of the mind as not to know the nature and quality of the act he was doing, or if he did know it, that he did not know he was doing wrong." *Second*, "That if the delusion under which the person labored was only partial, the party accused was equally liable with a person of sane mind. If the accused killed another in self-defense he would be entitled to an acquittal; but if the crime was committed for any supposed injury, he would then be liable to punishment."

The learned barrister (our author) would here come, doubtless, to a very different conclusion with reference to these tests of responsibility had he received his impressions of the motives and impulses, tastes and sentiments, intellection and perception of the insane from personal association with them, and the pertinacity he now displays in defending them would have been employed in proclaiming their unreliability. This association would have taught him that insane people in hospital do acts, that are unquestionably the offspring of disease, with deliberation, without the compulsion of irresistible impulse, which they know and admit to be wrong, and for which they would tell him that they deserve punishment. He would moreover have ascertained that there is no such thing as partial delusion, and that the fact of the existence of a delusion unquestionably implies such a serious error in intellection as would prevent a man reasoning with the same correctness of judgment as sane men do, and that it is therefore wrong to bind him down to the same tests.

The whole difficulty, in our opinion, arises from the failure of our author and the judges to consider mind as a totality. It has been aptly compared to a delicate machine, every part of which is essential to perfect work, and in which the slightest derangement of any part produces disorder and confusion.

In our opinion there are but two questions to be given to the jury in cases involving responsibility: *First*, Is the prisoner insane; and, *Second*, Is the alleged criminal act the offspring of disease. These being determined in the affirmative, the prisoner should be relieved of all responsibility.

Our author, like the legal fraternity generally, has not a very high appreciation of medical expert testimony. We are disposed to find fault with him for this, but nevertheless think his criticism is in part deserved. The medical profession at large has paid but very little attention to the study of insanity, and in addition, labor under the disadvantage of being summoned as experts by one side or the other in the suit, and frequently find themselves almost irresistibly, it may be, in the position of partisans. This last difficulty might be obviated by making the expert an officer of the court, summoned by the court, and under no obligation to either plaintiff or defendant.

We do, in conclusion, commend the book to all members of the medical and legal professions who are interested in this branch of medical jurisprudence. D. R. B.

A Practical Treatise of Diseases of the Eye, by *Robert Brudenell Carter*, *F.R.C.S.*, Ophthalmic Surgeon to St. George's Hospital, to the Royal South London Ophthalmic Hospital, etc. Edited, with additions and test-types, by Dr. John Green.

A notice of the English edition of Mr. Carter's work has already been placed before the readers of this journal.

We now welcome the American reprint, prepared by Dr. Green, of St. Louis, with his valuable annotations and additions.

An author who has enjoyed an extended and abundant experience, both in private and hospital practice, and who is a thorough master of the theory and practice of his specialty, can scarcely fail to produce a valuable work; as such we consider this volume.

We cannot but regret, however, that the author did not make so good a work, in some respects better than it is, and that the editor, so eminently qualified, did not increase the number and extent of his remarks and criticisms.

Such a treatise as this is intended principally for the use of beginners and for general practitioners.

Although the latter class, in country practice, can scarcely hope to become "ophthalmologists," they should qualify themselves for the skillful treatment of the various forms of external inflammation and iritis, and should be able to diagnosticate with the ophthalmoscope incipient cataract, intraocular tumors, adhesions of the iris and lens.

The faithful study of Mr. Carter's work will enable any careful practitioner, who has attended an eye clinic during his college course, to accomplish this. The various subjects are usually discussed in clear language, which cannot fail to be understood.

Technical terms are almost invariably explained in the text, and yet we believe a glossary of words, frequently found in ophthalmic literature, would be a great convenience to the young student, if not to the old.

For instance, we think it would greatly puzzle a student reading page 207 of Well's text book, to discover the true meaning of "Cataracta accreta." To what book could he turn for a solution of the mystery.

The index is somewhat imperfect. The index of any work worth reading can scarcely be overcrowded with minutiæ. A glance down the columns of an index is a review of the whole work; it reminds the student of those subjects of which he is still ignorant, and which he needs to reconsider.

We can commend the manner in which drawings of instruments are made, and in which are given their names based on their use, or the names of their inventors. The terms applied to operations and formulæ of remedies, based on the names of their originators, are also generally well explained.

The subject of the conjunctivitis of new born children is dismissed with a notice too brief. While we entirely agree, with the author, in regard to the treatment in almost all cases, by means of mild astringents, frequently applied, we do not believe those cases, not altogether exceptional, in which, early in the disease, there is great swelling of the conjunctiva and lids, and great secretion of pus, can be safely entrusted to the care of ordinary nurses and parents. In such cases our experience places us among those who trust to quite strong solutions of arg. nit applied evenly but not too freely over as much of the conjunctiva as possible. This cannot be properly done by a nurse inexperienced in everting swollen lids.

It is not especially rare that a mother brings to the surgeon her babe, and with it a little crystalline lens carefully folded in a paper. We doubt whether, in such a case, Mr. Carter, with all his skill and gentleness, could be sure he could separate the lids of the other eye without danger of seeming to observing friends to "squeeze" out the lens.

The few paragraphs devoted to this subject, read before a jury trying a physician for malpractice, would not be to the advantage of the defendant, however innocent he might be of mismanaging the case.

The physiology of the superior and inferior recti and of the oblique mucles, should, we think, have been concisely explained, for nearly all our standard works on anatomy and physiology are singularly at fault on this subject.

We are glad to observe an allusion to the fact, mentioned in scarcely a work in English, that calomel should not be dusted into the eye when iodide of potash is taken internally, on account of producing irritation or even cauterization of the conjunctiva.

It is worthy of notice that the author frequently gives ample credit to American specialists.

We repeat that, except for a few faults, which we consider, however, as quite grave, we highly commend this work, and believe its clear description of disease, and its lucid explanation of the methods of diagnosis and treatment will soon render a new edition necessary. E. L. H.

De la Valeur de l'Hystérotomie dans le Traitement des Tumeurs de l'utérus. Présentée au concours pour l'agrégation (section de chirurgie et d'accouchements) et soutenue à la Faculté de Médecine de Paris par le *Dr. Samuel Pozzi*, Ancien aide d'anatomie à la Faculté, Lauréat des Hôpitaux (prix des internes, médaille d'or,) Membre de la Société d'Anthropologie, etc. Paris, 1875.

In the year 1873 there appeared a remarkable little book, a translation of the title of which is, "*Hysterotomy for the*

*ablation (partial or total,) of the Uterus by Gastrotomy. A study of those tumors which may necessitate that operation,*" by G. Pean and L. Urdy. It was published in Paris. The object of it was to advocate gastrotomy as one of the justifiable measures in the treatment of fibrous tumors of the uterus under circumstances where no other remedy seemed available. The work made a profound impression on the profession of the whole civilized world, and was a necessary predecessor to the thesis of Dr. Pozzi.

In the work of MM. Pean and Urdy, M. Pean informs the profession that he had performed the operation nine times and met with success in seven of his cases; at the wonderful rate of seventy-eight per cent. of cures. Dr. Pozzi, in his thesis, gives first the history of the operation, from which we learn that all the first operations were begun and many of them finished under the impression that the operator had to deal with an ovarian instead of a uterine tumor. The first of these mistakes occurred in the hands of Dr. Charles Clay, of Manchester, England, which was soon repeated by Mr. Heath of the same place.

Dr. Burnham, of Massachusetts, seems to have been the first to meet with success. His operation was performed in June, 1853, and published in *Nelson's American Lancet.* After this Peaslee, Kimball and Boyd, of this country had successful blunders of the same kind. Then came Spencer, Wells and Kœberle. The first person to advocate and perform gastrotomy for fibrous tumors of the uterus was Kœberle, of Strassburg, 19th of December, 1863. He was closely followed by Dr. Horatio Storer and several European authors. The French Academy of Medicine, and the large body of the profession at that time declared against the operation. We think now, however, that there is a strong disposition on the part of the ovariotomists, if not of other surgeons, to place this operation on the list of legitimate and justifiable measures in certain desperate cases. Dr. Pozzi says the operation (gastrotomy) should be reserved for such cases of fibrous tumors or fibrocystic tumors of the uterus of rapid, galloping growth, as are accompanied by grave symptoms which threatens the life of the patient.

The fatality of the operation was very great at first when it was merely stumbled upon, and performed hap-hazard; but it would seem from the success of M. Pean that when deliberately entered into, and skillfully executed, the dangers from the operation is not greater than in ovariotomy. Dr. Pozzi publishes a table in which he has collected 119 cases, including the accidental as well as the deliberate operations, and the mortality amounts to about 67 per centum.

Every one knows how to estimate the results of a new operation, performed by all sorts of surgeons, under the adverse circumstances of being unprepared for it, the operator not suspecting the nature of the tumor, and entirely inexperienced in the method of removing it, and the care of the patient afterwards. The only wonder is that the mortality was not greater. It would seem that we really have no other series of cases than those presented by M. Pean upon which to base an impartial judgment.

The cases of M. Pean, as given in this thesis of Dr. Pozzi, in 1875, present to us a remarkable amount of success for any capital operation. In twenty-four cases there were eighteen recoveries and six deaths. Seventy-five per cent. of his cases were therefore successful. This would be a good showing for ovariotomy.

In addition to the subject of abdominal hysterotomy, Dr. Pozzi investigates in a thorough manner the removal of polypi by dilatation of the cervix uteri, enucleation, intra-uterine hysterotomy, retro-uterine hysterotomy, and amputation of the inverted uterus containing fibrous growths. His researches are extensive and very interesting to the gynecological reader especially. It would hardly be profitable to follow him through all these various divisions of his thesis in a journal like ours. In fact the object had in view in this notice of the work under consideration was to present to our readers an idea of the rapid advancement toward perfection of the momentous operation of abdominal hysterotomy.

In perusing the rapid sketch we have here given of the present status of abdominal hysterotomy, the senior reader will hardly fail to see a close parallel between its present condition and ovariotomy twenty-five years ago. In speaking of the first cases as blunders, mistakes, etc., we do not wish to be understood as treating irreverently the great men who happened into them. It will be remembered that our means of diagnosis of abdominal tumors — not yet perfect — are now so much in advance of what they were at the time these mistakes were made that we cannot wonder at them. Our astonishment will be very much modified, also, from the consideration that among the men thus failing in their diagnosis were Spencer Wells, Atlee, Kimball, Kœberle, and others.

With the means now at hand for distinguishing between these morbid growths, such mistakes, if they occur at all, will be very few indeed. Hence we may assume that in future a more deliberate and judicious experience will very soon enable us to arrive at just results.

A Manual of Diseases of the Eye, by *C. Macnamara*, Surgeon to Westminster Hospital. Third edition. Lindsay & Blakiston, Philadelphia. 1876.

A careful perusal of this work convinces us that it is an excellent manual of eye diseases. The author presents in a small volume, and in a clear and well arranged manner, a pretty complete account of modern ophthalmology.

The work is divided into sixteen chapters. The first and second treat of the anatomy, accommodation and refraction of the eye; chapters three, four, five and six treat of the diseases of the orbit, lids, lachrymal passages and sclerotic; the seventh, eighth, ninth and tenth are on the diseases of the conjunctiva, cornea, iris and choroid; chapters eleven, twelve and thirteen treat of the diseases of the retina and optic nerve, vitreous and lens; chapter fourteen of affections of the muscles; chapter fifteen of the disorders of refraction and accommodation; chapter sixteen is a brief notice of congenital malformations and diseases of the eye. While the author as a rule coincides with the most generally accepted views on eye diseases, and gives to each disease about the amount of space its importance demands, we think he varies from this in some instances.

In the third chapter, regarding the treatment of scirrhus of the orbit, he says: "I am opposed to surgical interference; the use of the knife I believe accelerates its progress, and the chances are infinitely small of our being able to eradicate the cancer." We agree with him that the chances are small, but even an infinitely small chance is better than none at all. He refers to a case reported by Mr. Lawson, which, after a thorough removal of the growth, progressed favorably, and eleven months afterwards presented no signs of the return of the disease, and says: "Cases of this kind seem hopeful, nevertheless they do not deter me from pressing my opinion as to the advisability of non-interference with the knife in scirrhus of the orbit." Of two evils we prefer the least, and must dissent from this view.

Diseases of the lids, lachrymal passages and sclerotic are treated of in a concise and practical manner. The various inflammations of the conjunctiva are considered under the following heads: hyperæmia, muco-purulent, purulent, diphtheritic, granular and pustular conjunctivitis. Infantile and gonorrhœal conjunctivitis are included (and we think properly) under the general head of purulent conjunctivitis.

In a note on page 159 the author says: "It seems to me hardly wise to retain the word ophthalmia to designate diseases of the conjunctiva. We employ the terms iritis and choroiditis and so on to signify inflammation of the iris and choroid; why not, therefore, conjunctivitis in analogous diseases of the conjunctiva." We echo the question, "Why not?" This term seems to us to be the simplest and most scientific, and it is certainly desirable to have uniformity in the nomenclature of eye diseases. Iritis is described under three heads: plastic, serous and parenchymatous. With regard to the use of mercury in the treatment of iritis, Mr. M. says: "As a general rule it may be affirmed that mercury is rarely to be employed in iritis, unless in cases arising from syphilis; in other instances of iritis this drug is seldom required." With this view we heartily concur.

Glaucoma, which is considered in the chapter on the diseases of the choroid, is not given the space its importance demands, as it is dismissed with seven or eight pages. With regard to the treatment of glaucoma, the author says: "There can be no doubt whatever that iridectomy, if practiced sufficiently early, will cure the disease" — "the operation consists in the successful removal of a section of the iris, together with its ciliary attachments."

Mr. M. makes an uncalled-for digression in chapter eleven, where he describes as hyperæmia of the retina what is properly described by other authors as serous retinitis. Glioma is dismissed with less than three pages. The author must have a view of his own about the malignancy of morbid growths. Disregarding the clinical facts of secondary tumors in the liver and other organs observed in numberless cases of glioma, he surprises the reader with the strange statement: "These tumors are of a slow growth, and it is doubtful if they are malignant."

The author's long residence in India has afforded him valuable experience upon the influence of climate, race, malarial poison, etc., in the production and treatment of some eye diseases.

The volume is supplied with marginal notes on its contents, which will greatly facilitate reference; but it is questionable whether they are profitable to the student. The text is fairly illustrated with cuts and diagrams. There are also a number of chromo-lithographic plates, which are far from being creditable. Most of the figures are copies from other works, but are miserable representations of what they are intended to illustrate. Notwithstanding these minor defects, the work is one that we can heartily commend to the student and general practitioner, and as worthy of a place in the library of the specialist.

W. T. M.

LECTURES ON ORTHOPEDIC SURGERY AND DISEASES OF THE JOINTS. *By Lewis A. Sayre, M.D.* Published by Appleton & Co. New York, 1876.

The preface states that these extemporaneous lectures are printed without change, exactly as taken down by a stenographer attending for the purpose.

This can hardly be true in the strict sense, as the first lecture would occupy only fifteen minutes, and the second one only nine minutes in delivery, and there are no clinical cases introduced in either whose examination could account for the remaining part of two ordinary lecture hours. We must suppose that the statement of the preface is an inadvertancy, and that the author, like other sensible men, revised and rearranged the first notes so as to present the subject matter in proper book form.

The first lecture is devoted to a historical and general discussion. The second refers very briefly to the definitions and etiology of deformities. The third lecture takes up the topic of *Phimosis* as the cause of infantile paralysis, and gives some remarkable cases of cure by circumcision. This is perhaps one of the most singular and valuable discoveries in modern minor surgery, if further experience shall fully confirm it. The notice of it in these lectures is, however, very brief, and the reader will need to complete his study of the topic by the the help of medical journals and society transactions of the last three years.

Lectures four, five and six discuss very briefly some of the practical measures applicable to deformities in general, such as tenotomy, electricity, mechanical appliances, gymnastics, etc.

The next five lectures are devoted to the important subject of Talipes. They are copiously illustrated, and while there can be no claim to completeness in a discussion of talipes, occupying only ninety pages, yet there is so much that is practically useful condensed into them that they are worthy of high commendation.

Lecture twelve treats of corns, bunions, bow-legs, knock-knee, etc. Lectures thirteen to twenty-four give us the author's views on diseases of the locomotor joints, commencing with the ankle. For extension on inflamed ankles Sayre still uses his somewhat cumbrous and expensive ankle-extensor.

It appears to us that the neat and simple leather apparatus commonly used in Chicago, which is moulded on a plaster cast of the foot, and acts by pressing the foot down and the leg up, on the principle of two hollow cones apex to apex, is much preferable.

Sayre introduces here a very ingenious method of making extension on inflamed tarsal joints. This consists in placing five "Indian puzzles" on the toes, and by means of them suspending the foot to a cross-bar.

The knee joint is next taken up, and the pathology of its diseases briefly stated. On page 202 is depicted a method of making extension in bed on a knee which is both inflamed and bent, so that extension cannot be made in the straight position. It consists in an ordinary stretch on the leg by adhesive straps, weight and pulley, to which is added a simple band under the calf of the leg, pulling obliquely upward and toward the foot, so as to hold the knee bent at any desired angle. Sayre seems unacquainted with the simple and efficient apparatus used by Chicago surgeons for knees which are bent and at the same time inflamed, and which allows the patient to go about on crutches; for he says the "extension must be made while the patient is in bed until the limb is brought nearly to a straight position before the instrument (his straight extender) is applied." His ingenious and compact extender is then described, and it is certainly worthy of high praise in cases where the limb is straight. In our opinion, however, the extension apparatus of the late Prof. Sherman, of this city, is equally efficient, and has the merit of being capable of being put on and taken off with much less trouble. Sherman's instrument also rarely causes the swelling of the knee, which Sayre says is produced by the constriction of his apparatus if the knee-bandage is omitted. Yet we think Chicago surgeons are very negligent, in the fact that they do not more generally apply the knee-bandage in cases of instrumental extension, and they would do well to give heed to the author's precepts on that point. His words are, "compression is an essential element in the management, and must never be neglected."

After the diseases of the knee, the lectures proceed with *Morbus coxarius* and spinal diseases, but the limits of this notice do not permit of a full abstract of them. As a whole they are interesting, and contain many practically useful precepts, though, being only clinical lectures, they are necessarily somewhat desultory and incomplete, and make no pretense of being a systematic treatise. E. A.

Transactions of the Pathological Society of Philadelphia. Volume Fifth. Edited by *James Tyson, M.D.*

This most valuable volume of a most industrious society contains the material contributed during the whole of 1874 and the first part of 1875. The volume reflects great credit upon the Pathological Society, and especially upon the editor. The book is made up almost wholly of descriptions of pathological specimens. The list of them is as follows: Thirteen of the osseous system; thirty-four of the digestive apparatus, including ten of the liver and its appendages; twenty-five of the vascular system; thirteen of the respiratory system; twenty-four of the genito-urinary apparatus; eight of the nervous system; five of the organs of special sense; five of diseases of the ductless glands; twelve tumors not otherwise classified, beside a half dozen or more of miscellaneous specimens.

Twenty-two specimens were referred to a committee on morbid growths, whose reports upon the cases are published. The work is embellished with sixteen wood-cuts and is supplied with a very full index. The histories of specimens are brief, concise and evidently accurate, and are only accompanied by a sufficient report of the remarks made in the Society at the time of presentation to make the whole account pleasantly readable and thoroughly understandable. In the space at our command it is impossible to give anything like a complete idea of the contents of the volume; it would require many pages to do this. But we heartily say it is one of the most interesting and valuable records of pathological history — the history of pathology — it has been our fortune to examine. Such records of medicine, becoming in the years a great wealth to the profession, cannot be too highly commended nor too warmly encouraged. We are only pained that some cities we could mention, west of Philadelphia, and nearly as large, have so far shown no promise of similar efforts.

Medical Diagnosis with Special Reference to Practical Medicine. A guide to the knowledge and discrimination of Diseases. By *J. M. Da Costa, M.D.*, etc. Fourth edition, revised. Philadelphia, J. B. Lippincott & Co. 1876. pp. 835.

We always, since the appearance of the first edition of this book, have regarded the work of Da Costa as one of the most valuable of American contributions to the study of human disease. We have had in this belief, we are sure, the almost unanimous agreement of those of the profession who have been familiar with our author's work. The book has been during the last dozen years really and safely "a guide to the knowledge and discrimination of diseases" to many of us groping and often puzzled practitioners.

The American profession know very well the use to which Prof. Da Costa has put his time and opportunities during these years, and do not need to be told that, in the numerous additions and changes incorporated in this revised edition, the work has been made still more worthy of their confidence. The changes and additions show themselves most in the parts devoted to nervous diseases and fever, two classes of affections which have of late engaged so much study and attention among our best students. We do not believe the whole is yet known about these disorders, and have a strong suspicion that not a little will have to be unlearned in the future, but Da C. has endeavored to give us safe rules for practical guidance, and we have no doubt he has succeeded as far as is in this day possible.

We have not space to note the particular changes which this edition shows from the former; they are both numerous and valuable. All books have faults; this one has, but it has many virtues, and is altogether the most trustworthy and valuable on the subject of which it treats of any the American student can get hold of. It ought to form a part of the library, of many more physicians than it has heretofore.

Transactions of the Twenty-fifth Anniversary Meeting of the Illinois State Medical Society, held in the City of Jacksonville, May 18, 19 and 20, 1875; 8vo., pp. 288. Chicago: Fergus Printing Company, 1875.

The first article, of general interest, is a report on scarlatina, by Dr. J. P. Walker, presenting a new remedy and prophylactic, viz.: iodide of potassium. He claims to have treated two hundred and fifty cases of varying degrees of severity, without a fatal result where the method was properly carried out. He insists on free greasings from the first, and with the drug named, gives scilla and veratrum viride. We hope a more extended experience will confirm the satisfactory results he has obtained.

The second is a report on the anatomy of serous membranes by Dr. Lester Curtis, and is an entertaining effort to explain the cause of the adhesions that follow the inflammation, particularly of the pleura and peritoneum.

There is a report on the treatment of whooping cough, by Dr. J. O. Hamilton, supplemented by a report on the same subject, by Dr. Chas. W. Earle. These reports give the experience of the authors in the use of quinine in this disease, and fully confirm the observation made and reported a few years ago by Dr. B. F. Dawson, of New York City. The case reported by Dr. Earle is novel, inasmuch as the quinine was locally applied with the atomizer. A limited experience we have had with this remedy has been as satisfactory as that detailed in these reports.

The paper on transfusion of blood, by Prof. J. W. Freer, is an able account of the history and method of using this recently revived therapeutic agency. He expresses his preference for the indirect method, and describes a very simple apparatus for the purpose. Speaking of transfusion in chronic cases he says: "In this manner, for the time being, the means are furnished for a more or less successful warfare against disease and natural waste; but if the digestive organs do not, in the meantime, profit by this respite, and regain their normal power and activity, the tissues and organs must soon again be on short rations, while the patient naturally sinks to the old standard." Speaking of it in danger from acute hæmorrhage, he says: " So certain is trasfusion of normal blood to resuscitate and restore to life, that we feel warranted in asserting, emphatically, that the practitioner in charge is under the most sacred obligations to perform this operation of transfusion." An account is given of experiments by himself that seem to show that blood retains its nutritive and vivifying properties, for some time after being drawn, for the living tissues. In one case the blood was not transfused until seventy-two hours after it was drawn, and the operation was successful.

The next in order is a report on practical medicine, by Dr. E. P. Cook, and a contribution to the same, by Dr. R. C. Hamill. The first is entitled to consideration especially because it recommends a return to the old treatment of blood letting in pneumonia and pleurisy; the second is important because it presents to us a combination of remedies that, in the doctor's hands, has been very successful in the treatment of sub-acute and chronic rheumatism and in tonsillitis. This combination consists of equal parts of tinctura phytolaccæ and tinctura guaiaci. The author also recommends these remedies in certain forms of dysmenorrhæa, in migraine and the latter remedy in scarlatina and diphtheria. We doubt very much whether these remedies will be attended by any such good results in the hands of the profession at large, for we are of the opinion that no little of the success is due to the personal magnetism and will power of the doctor himself.

The report on gynecological instruments, by Dr. T. D. Fitch, shows that the inventive genius of the State has been very active. Three new speculums, a new vaginal retractor, a new tenaculum, a new uterine sound and uterimeter and a new pessary have been recently added to the uterine armamentarium.

The report on obsetrics, by Dr. J. B. Rood, deals especially in post-partum hæmorrhage and enumerates, as valuable agents for its relief, injections of perchloride of iron, injections of hot water, and the electro-magnetic current. An account is also given of three cases of arm presentation, in which he successfully practiced cephalic version.

The report on small pox, by Dr. E. W. Gray, is a very able resume of the etiology, pathology and prophylaxis of this loathsome disease. The fact is developed that there is wide spread neglect, throughout the State, of vaccination, and, as a remedy, our author very wisely demands legislative interference in the shape of compulsory vaccination for the protection of the people. He sagely remarks: "that every life lost by this disease is needlessly sacrificed, and that a matter of such gravity should not be left subject to individual caprice and folly." We trust his efforts may be successful.

In conclusion, we wish to express the great pleasure we have derived from our perusal of the Transactions, and congratulate the Secretary and society generally on the many excellencies of the volume. D. R. B.

CHIRURGIE ANTISEPTIQUE, PRINCIPES MODES D'APPLICATIONS ET RESULTAT DU PANSEMENT DE LISTER. Par Le *Dr. Just. Lucas-Championnière.* Paris, Baillière et Fils. 1876.

Since the readers of this journal are (or at least ought to be) well acquainted with the so-called antisceptic treatment and with Lister's essays on this subject, the review of the book before us can be very brief. It is a French reiteration of Lister's doctrines. The writer explains and ably argues the germ theory on which the antisceptic treatment is based, he minutely describes all the details and variations in the mode of antisceptically dressing a wound, and enumerates some of the most brilliant results obtained by this treatment. To become thoroughly familiar with the antisceptic treatment, Dr. Ch. spent some time in Edinburgh with Lister. He returned to France an enthusiastic admirer of Lister's doctrine, a firm believer in the antisceptic surgery which to introduce into the French hospitals then became the chief object of his life.

We congratulate both Mr. Lister and the French profession. The former could not have found a more devoted and zealous apostle for preaching his principles in France, and they could not wish for any better information about the antisceptic surgery than which is condensed by an elegant writer in this little volume. The pleasure we enjoyed in reading it would have been complete if only the Continental publishers could be induced to print their books on good paper, and to send them out into the cold world dressed, cut and bound.

CONSUMPTION IN AUSTRALIA. *By C. E. Reeves, B.A., M.D., etc.* Melbourne. 1874.

This is a little book of 154 pages, in which the author discusses very clearly and fairly this disease as it occurs in that far off country. It is not a book on consumption as a disease, so much as it is a book on this disorder as it occurs in Australia. In treating of the influences that bring on consumption he says the deposit of tubercles is very rare. Most of the cases are inflammatory in their nature. The deposit of tubercles in other organs than the lungs is rare. He thinks the climate is more favorable for invalids afflicted with this malady than that of England.

THE STUDENT'S GUIDE TO HUMAN OSTEOLOGY. *By W. W. Wagstaffe, B.A., F.R.C.S., etc.* 12mo., pp. 349. Philadelphia. Lindsay & Blakiston. 1875.

The profession is to be congratulated on the appearance of any creditable book that will aid in the study and understanding of anatomy. Such a book is the little volume before us. It accomplishes one aim of the author, "to describe the bones of the human skeleton with accuracy, but without wordiness," very fully.

All the bones of the skeleton are in their outward appearance carefully described, mostly after the plan usually followed in such descriptions.

The account of the mechanical structure is made up to a large degree of the author's own observations. The book contains twenty-two double page lithograph plates of the bones, with red line boundaries of the insertion of muscles, besides many wood-cuts illustrative mainly of the mechanical structure of the bones and their development.

Very few errors, we are sure, can be found in the volume; so few we are not disposed to undertake the task of noting them.

INHALATION IN THE TREATMENT OF DISEASE: ITS THERAPEUTICS AND PRACTICE. A treatise on the inhalation of gases, vapors, fumes, compressed and rarified air, nebulized fluids and powders. By *J. Solis Cohen, M.D.*, etc. Second edition revised and enlarged, with many new illustrations. Philadelphia: Lindsay & Blakiston. 1876.

The title of this book suggests its wide scope. A careful examination of the text does not disappoint the reader. Dr. Cohen has made his work thorough and searching in every department. The book is divided into four parts. In the first the inhalation of airs, gases, vapors and fumes is considered; in the second the inhalation of nebulized fluids or sprays, and in the third the inhalation of powders. The fourth is devoted to a brief consideration of medicated atmospheres.

Under these several heads there is gathered an amount of information on the subjects considered that is not approached by any book we know of in the English language. Many writers in several languages have been laid under contribution; the periodical literature has been searched and used, and the whole work has been shaped and enhanced by the experience, the scholarship and good sense of this industrious American author.

There has been of late no little nonsense, both in and out of the profession, on the subject of inhalations. It has been the net by which many quacks have captured people — or their money, not often their diseases; it has been the pivot on which not a few previously very sane doctors have whirled until they were more than dizzy. The therapeutical value the measure possesses is not so great as the enthusiasts believe; it is far greater than the average practitioner has any idea of. Dr. Cohen has ably contributed to the placing of the subject on a basis of science for which he is honestly entitled to the thanks of the profession.

